# When a calf mass is not a tumor: dynamic ultrasonographic diagnosis of an accessory soleus muscle

**DOI:** 10.12701/jyms.2026.43.56

**Published:** 2026-08-18

**Authors:** Wei-Ting Wu, Ke-Vin Chang, Levent Özçakar

**Affiliations:** 1 Department of Physical Medicine and Rehabilitation, National Taiwan University Hospital, Bei-Hu Branch, Taipei, Taiwan; 2 Department of Physical Medicine and Rehabilitation, National Taiwan University College of Medicine, Taipei, Taiwan; 3 Center for Regional Anesthesia and Pain Medicine, Wanfang Hospital, Taipei Medical University, Taipei, Taiwan; 4 Department of Physical and Rehabilitation Medicine, Hacettepe University Medical School, Ankara, Türkiye

A 54-year-old woman presented with a 3-month history of a palpable mass on the lateral aspect of her left calf. The mass was painless and only noticeable during toe walking or maximal plantar flexion. No visible or palpable mass was observed at rest. Furthermore, the borders of the lesion were indistinct and could not be clearly delineated by palpation. Because of these unusual features, the patient was referred to our physical and rehabilitation medicine clinic for further evaluation. Her medical history was otherwise noncontributory. Physical examination revealed no obvious swelling while the patient was standing in a relaxed position. However, during toe walking or maximal plantar flexion, a focal soft-tissue prominence was evident over the lateral aspect of the left calf (Fig. 1A). The protrusion disappeared immediately after the ankle was returned to a neutral position.

Ultrasonography revealed an additional muscular structure in the soleus region. Compared with the surrounding soleus muscle, this structure appeared relatively separate from the main muscle belly and was slightly more hyperechoic (Fig. 1B, Supplementary Video 1). Examination of the contralateral calf revealed no comparable structure (Fig. 1C, Supplementary Video 2). During active plantar flexion, the accessory muscle contracted synchronously with the adjacent soleus muscle, and its fibrillar echotexture (Fig. 1D) was identical to that of normal skeletal muscle. Longitudinal ultrasound tracking from the proximal calf toward the ankle further confirmed that the soleus at the target site did not extend to the lateral ankle. Moreover, no comparable muscle belly was identified at the corresponding level of the contralateral calf, making a low-lying soleus less likely. These findings were consistent with those of an accessory soleus muscle, rather than a soft-tissue tumor or other pathological masses. The patient was reassured that no treatment was required and was advised to return for reassessment if she experienced pain, progressive enlargement of the mass, or functional limitations.

An accessory soleus muscle is a rare congenital anatomical variant with a reported prevalence of approximately 0.5% to 6% [1,2]. It is usually asymptomatic and often discovered incidentally. Symptomatic cases generally occur in young, physically active individuals, and are attributed to exertional compartment syndrome, localized ischemia during exercise, or tibial nerve compression that causes tarsal tunnel syndrome. Unlike previously reported cases, our patient did not present with the characteristic posteromedial ankle swelling. Instead, the mass was located in the middle portion of the calf and visible only during muscle contraction. Most published cases [1,2] describe the accessory soleus muscle near the ankle (i.e., soleus tertius), where the normal soleus has already transitioned into the Achilles tendon, making the accessory muscle belly readily apparent. Therefore, our case expands the clinical spectrum of the accessory soleus muscle by demonstrating that a more proximally located accessory muscle may present as a dynamic midcalf mass, making clinical diagnosis considerably more difficult. Furthermore, dynamic ultrasonography demonstrated that the mass had close anatomical continuity with the soleus muscle and was located relatively superficially within the posterior compartment of the leg. These findings indicate that an accessory flexor digitorum longus is less likely. In addition, an accessory flexor digitorum longus typically courses distally toward the tarsal tunnel [3], where it is commonly identified as an accessory structure adjacent to the flexor digitorum longus tendon. In contrast, no extension toward the tarsal tunnel was observed in our case, further supporting the diagnosis of an accessory soleus muscle.

This unusual presentation can be explained by the complex architecture of the soleus muscle. A recent three-dimensional anatomical study [4] demonstrated that the soleus consists of four distinct compartments (medial-anterior, lateral-anterior, medial-posterior, and lateral-posterior) rather than a single homogeneous muscle. In our patient, an additional muscle bundle appeared to represent an accessory compartment beyond this normal configuration. Its identical echotexture to the native soleus and synchronous contraction during plantar flexion strongly supports a congenital accessory muscle rather than a pathological lesion.

This case also highlights the diagnostic value of dynamic ultrasonography [5]. A focal calf bulge may raise the suspicion of a muscle tear, hematoma, fibrosis, scar tissue, muscle herniation, or soft-tissue tumor [6]. However, these conditions usually result in disrupted muscle architecture, abnormal echogenicity, or asynchronous movement. In contrast, the accessory muscle in our patient displayed preserved fibrillar architecture and contracted synchronously with the adjacent soleus, allowing confident differentiation from pathological conditions without invasive investigation.

Although magnetic resonance imaging remains the reference standard for confirming the presence of an accessory soleus muscle, dynamic ultrasonography provides several practical advantages [7], including contralateral comparison, real-time assessment during muscle contraction, and evaluation of muscle continuity. To the best of our knowledge, this is the first report to describe an accessory soleus muscle identified using ultrasonography at the middle calf rather than at the ankle. Recognition of this atypical presentation broadens the differential diagnosis of exercise-induced calf masses and demonstrates that dynamic ultrasound can accurately identify accessory muscular anatomy, thereby avoiding unnecessary concerns regarding soft-tissue tumors and preventing unnecessary diagnostic procedures.

## Figures and Tables

**Fig. 1.**
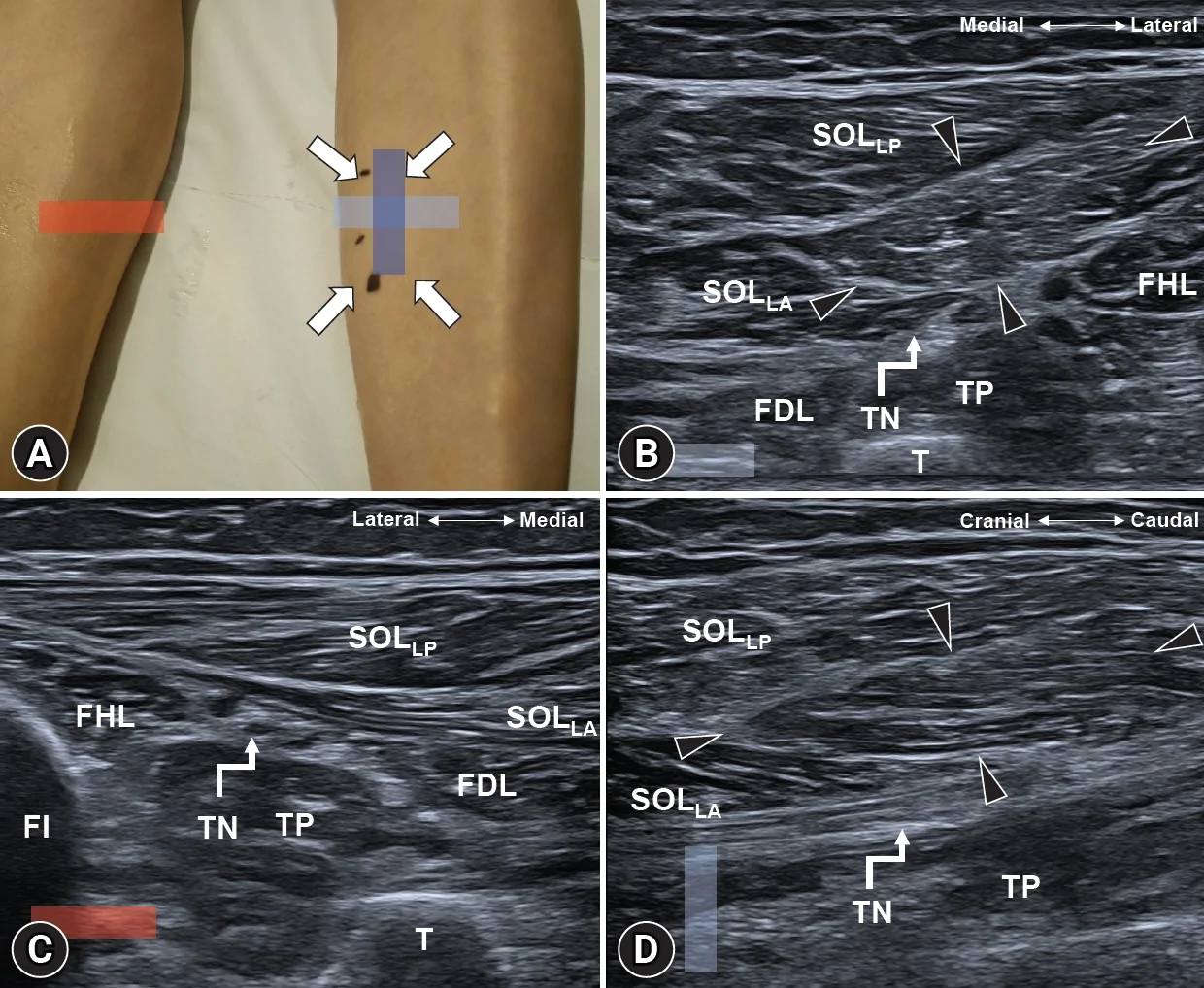
(A) Photograph demonstrating a focal soft-tissue bulge at the midcalf of the left leg (white arrows) that becomes evident during maximal ankle plantar flexion. (B) Short-axis ultrasound image of the left calf revealing an additional muscle structure (black arrowheads) adjacent to the normal lateral-posterior (SOLLP) and lateral-anterior (SOLLA) segments of the soleus muscle. (C) Corresponding short-axis ultrasound image of the right calf showing no additional muscle structure. (D) Long-axis ultrasound image of the left calf demonstrating that the accessory muscle structure exhibits the same echotexture as the adjacent soleus muscle, supporting its muscle origin. The colored squares indicate the transducer positions. TN, tibial nerve; TP, tibialis posterior muscle; FDL, flexor digitorum longus muscle; FHL, flexor hallucis longus muscle; T, tibia; FI, fibula.

## Supplementary materials

Supplementary Videos 1 and 2 can be found at https://doi.org/10.12701/jyms.2026.43.56.
